# Food restriction patterns and their association with growth and nutrition in Chinese pediatric atopic dermatitis patients

**DOI:** 10.1016/j.jdin.2026.06.023

**Published:** 2026-07-30

**Authors:** Lanning Xie, Zhenzhen Wang, Wenchao Li, Honghan Ge, Chuan Wang, Gongqi Yu, Gaoyuan Fan, Hong Liu, Furen Zhang

**Affiliations:** a School of Public Health, Cheeloo College of Medicine, Shandong University, Jinan, Shandong, China; b Dermatology Hospital of Shandong First Medical University, Jinan, Shandong, China; c Shandong Provincial Institute of Dermatology and Venereology, Shandong Academy of Medical Sciences, Jinan, Shandong, China; d School of Public Health, Shandong First Medical University, Jinan, Shandong Province, China

**Keywords:** atopic dermatitis, children and adolescents, food restriction, food sensitization, growth and development

*To the Editor:* Atopic dermatitis (AD) is a chronic inflammatory skin disease that affects up to 20% of children globally.^1^ While individualized avoidance of confirmed allergens may alleviate AD symptoms,^2^ the patterns of food restriction, the prevalence of food sensitization, and their association with growth in Chinese children with AD remain poorly characterized, warranting further investigation.

To address this gap, we conducted a real-world cross-sectional study at Dermatology Hospital of Shandong First Medical University (July 2024-July 2025). We enrolled 606 children (aged 2-18 years) with AD and collected data on dietary practices, anthropometric measurements, specific IgE (sIgE) levels, and biomarkers. Propensity score matching (PSM) was used to balance confounders for comparative analysis.

Among the 606 enrolled patients (Supplementary Table I, available via Mendeley at https://data.mendeley.com/datasets/ncz9sz48hd/3), 24.92% (151/606) reported food restrictions, whereas 30.20% (183/606) tested positive for sIgE. However, the alignment was poor: 46.36% (70/151) of restrictions lacked objective evidence, and 55.74% (102/183) of sensitized patients reported no food restrictions. Notably, the most frequently avoided foods-shrimp (16.50%), fish (16.01%), and crab (14.52%), categorized as “fa wu” (stimulating foods) in traditional Chinese medicine-differed markedly from the most common sensitizations: egg (14.69%), milk (12.05%), and wheat (8.58%) (Supplementary Fig 1, available via Mendeley at https://data.mendeley.com/datasets/ncz9sz48hd/3; Fig 1).^3^

**Fig 1:**
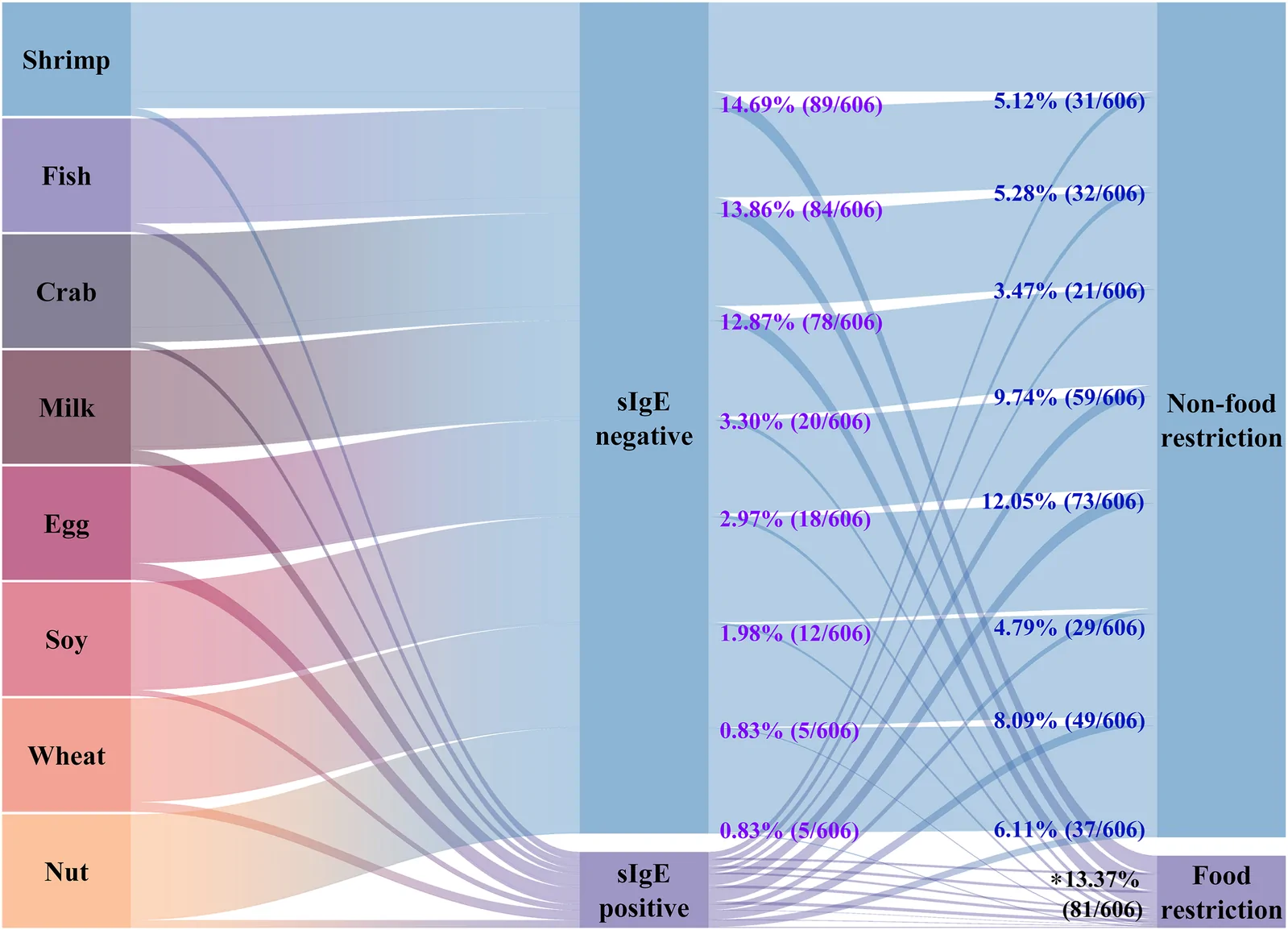
Discordance between common food sensitization profiles and patient-reported restrictions. The middle section categorizes participants based on food sensitization status, and the right section indicates the presence or absence of food restriction for each food. Connecting lines illustrate the distribution and correspondence between sensitization and restriction status across different foods. *Purple* counts represent the number and proportion of participants who were food sensitization-negative but still implemented food restrictions for various foods; *Blue* counts represent the number and proportion of participants who were food sensitization-positive but did not implement food restrictions for various foods. The *asterisk* (∗) denotes the number and proportion of individuals with positive food allergen test results among those on food restrictions. Food sensitization was defined based on specific immunoglobulin E (sIgE) testing toward food allergens. Serum allergen-specific immunoglobulin E (sIgE) levels were measured using the ImmunoCAP system (Phadia).

We then assessed the growth associated with these restrictions. After PSM, 336 matched subjects (112 with and 224 without food restriction; Supplementary Table II, available via Mendeley at https://data.mendeley.com/datasets/ncz9sz48hd/3) were analyzed. The restriction group showed lower height Z-scores (−0.11 vs 0.75), weight Z-scores (−0.03 vs 0.84), and BMI Z-scores (0.16 vs 0.63; all *P* < .001; Fig 2) than the non-restriction group, as well as lower serum albumin (45.60 vs 46.30 g/L, *P* = .022) and calcium (2.31 vs 2.41 mmol/L, *P* < .001) levels (Supplementary Fig 2, available via Mendeley at https://data.mendeley.com/datasets/ncz9sz48hd/3).

**Fig 2:**
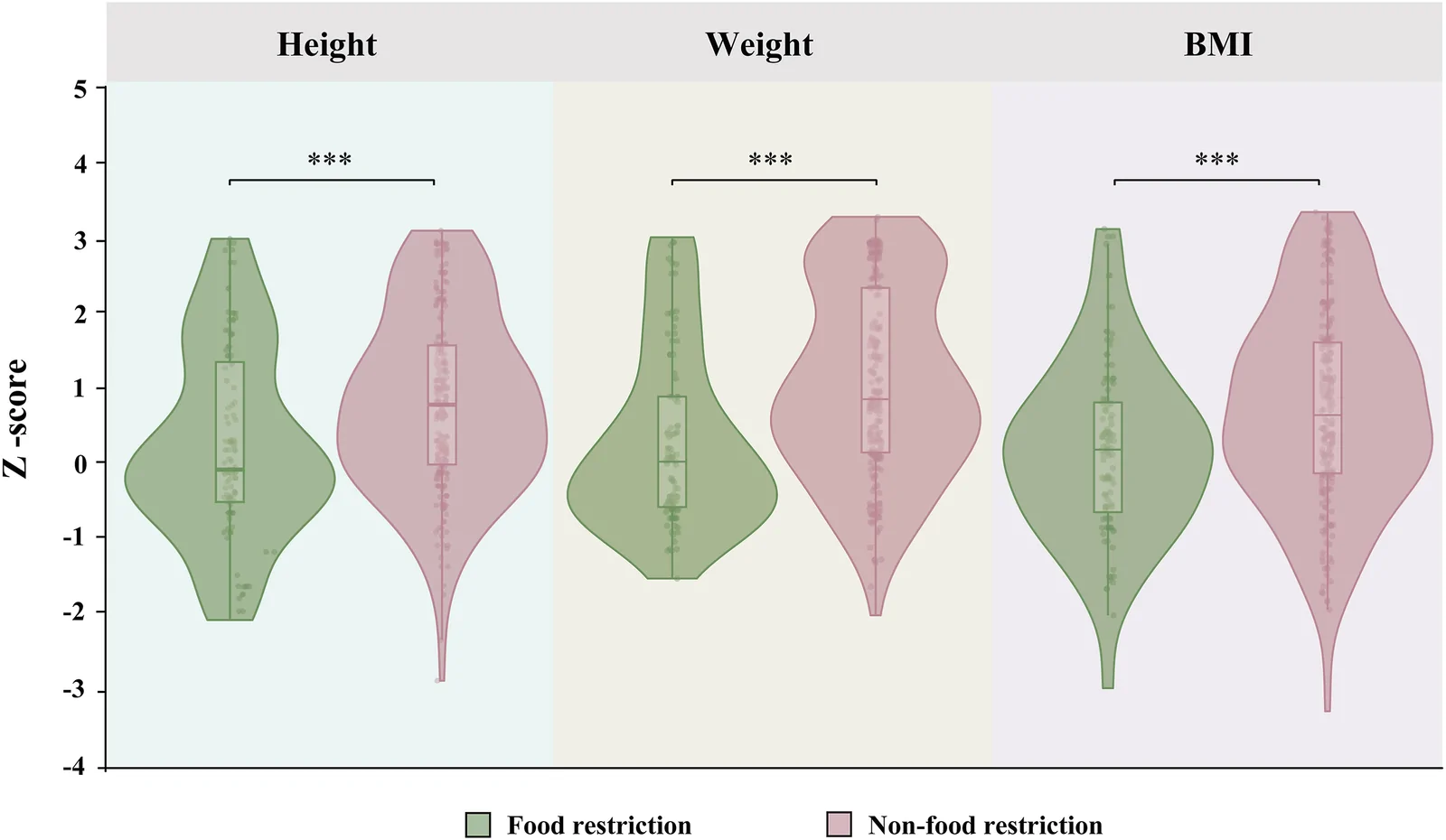
Association of food restriction with growth parameters in pediatric AD. Height, weight, and body mass index (BMI) Z-scores were standardized for age and sex. ∗∗∗*P* < .001.

To explore a balance between disease control and growth preservation, we conducted subgroup analyses based on restriction duration and the number of restricted food items. Restrictions lasting >3 months or involving ≥3 food items were associated with lower growth across all Z-scores (all *P* < .001). In contrast, restrictions lasting ≤3 months or involving ≤2 food items showed no statistically significant differences compared to the non-restriction group (Supplementary Fig 3, available via Mendeley at https://data.mendeley.com/datasets/ncz9sz48hd/3).

Finally, we explored the factors associated with the degree of food restriction. Multivariate analysis identified that food sensitization (OR = 4.16, 95% CI: 1.45-11.93), greater disease severity (OR = 5.90, 95% CI: 1.10-31.62), and longer disease duration (OR = 6.58, 95% CI: 1.70-25.42) were associated with extensive food restriction (Supplementary Table III, available via Mendeley at https://data.mendeley.com/datasets/ncz9sz48hd/3).

In conclusion, food restriction affected approximately one-quarter of this cohort, yet a substantial discrepancy exists with confirmed sensitization. Although indiscriminate restriction was associated with lower growth parameters, patients adhering to defined limits-specifically, avoiding no more than 2 food items or restricting for less than 3 months-showed growth parameters comparable to those without restrictions, suggesting that balanced management may be achievable. Comorbid food allergy, severe symptoms, or longer disease duration were associated with excessive restriction, highlighting the need for targeted nutritional monitoring.^4^^,^^5^

## Conflicts of interest

None disclosed.

## References

[1] Wang Z., Liang X., Li X. (2024). Global, regional, and national burdens of atopic dermatitis under 14: a trend analysis and future prediction based on the global burden of disease study 2019. Arch Dermatol Res.

[2] Kim S., Lee J., Ly S. (2016). Children with atopic dermatitis in Daejeon, Korea: individualized nutrition intervention for disease severity and nutritional status. Asia Pac J Clin Nutr.

[3] Souza Olegario L., Estevéz M., González-Mohino A. (2021). Cross-cultural emotional response to food stimuli: influence of consumption context. Food Res Int.

[4] Lim H., Song K., Kim R. (2013). Nutrient intake and food restriction in children with atopic dermatitis. Clin Nutr Res.

[5] Talamonti M., Galluzzo M., Silvaggio D. (2021). Quality of life and psychological impact in patients with atopic dermatitis. J Clin Med.

